# Serum zinc level and children`s asthma: A systematic and meta-analysis review article

**DOI:** 10.22088/cjim.12.3.236

**Published:** 2021-04

**Authors:** Javad Ghaffari, Reza Alizadeh-navaei, Abbas Dabaghzadeh, Negar Ghaffari

**Affiliations:** 1 Pediatric Infectious Diseases Research Center, Mazandaran University of Medical Sciences, Sari, Iran; 2 Gastrointestinal Cancer Research Center, Mazandarn University of Medical Sciences, Sari, Iran

**Keywords:** Asthma, Children, Zinc, Nutrition

## Abstract

**Background:**

Asthma is a chronic inflammatory respiratory disorder. Nutritional conditions affect allergic diseases such as asthma. The aim of this study was to review the serum zinc level in children with asthma.

**Methods:**

This is a review article found in databases such as Google, PubMed, SID, Irandoc, Scopus and up-to-date. Key words for search included zinc, asthma, children and pediatric. There was no time limitation for the search. These articles on zinc levels in asthmatic children were meta-analyzed.

**Results:**

Out of the 40 articles, 19 articles were excluded and 21 articles were included in this analysis. 15 articles evaluated serum zinc levels, 4 articles on hair zinc levels, one article evaluated nail zinc levels and another on zinc level in erythrocyte cells in children with asthma. Only 3 articles evaluated effects of zinc supplement treatment in children with asthma. Meta-analysis of studies showed that there was no significant difference between the standard mean differences of zinc level in asthmatic patients compared to the control group. We cannot analyze the association between zinc levels in hair and nail in children with asthma. All clinical trial studies show that zinc supplement improves clinical manifestations of asthma and patient’s pulmonary function test.

**Conclusion:**

We found that the mean serum zinc level difference is not significant in children with asthma than healthy control group and it seems that there is no relation between mean serum zinc level and severity of asthma in children.

Asthma is a chronic inflammatory disorder of the respiratory system, more common in children than adults. Asthma has different endotypes and phenotypes. Asthma in children causes significant morbidity and mortality. The disorder is the most common cause of emergency department visit, absence from school and hospitalization in children. The most common clinical manifestations of asthma are cough, wheezing and dyspnea. More than 300 million people are affected by asthma in the world. The prevalence of asthma varies in different areas (1, 2, 3). The prevalence of asthma has continuously increased from the past few decades, probably due to environmental and/or genetic susceptibilities, economic, and nutritional factors (4).The etiology of asthma is not exactly clear. Genetic and environmental factors have an important role in inducing and exacerbating asthma. Different cytokines and chemokines contribute in the pathogenesis of asthma (5-7). Diagnosis of asthma is based on patients’ history and physical examination. Spirometry should be done in more than 5 years old children suspected of asthma (1, 2).

Treatment of asthma includes avoiding trigger factors, pharmacotherapy such as short acting beta2 agonists (attacks); inhaler corticosteroids (choice for controller) and fewer cases need specific allergen immunotherapy (1). 

Incidence of asthma has increased due to the decreased amount of antioxidants in the diet in recent years (8). Trace elements such as zinc as an antioxidant may be effective on pathogenesis and severity of asthma. Zinc has antioxidant, anti-apoptotic and anti-inflammatory effects inhibiting NF-kB activation. Anti-oxidant substances inhibit production of free radicals such as reactive oxygen species (ROS), including superoxide and hydrogen peroxide (H2O2) (8). Zinc is an important trace element in immunological processes, oxidative stresses and the inflammatory reactions. The main oxidant factors are O 2, H 2 O 2, and OH (8). Zinc protects the respiratory system through its antioxidant effect, stabilizing microtubules, anti-apoptotic effect, and growth co-factor (8, 9). Zinc has an important role in the synthesis of DNA, RNA, energy production, protein and cell and tissue growth of cell. Low intake of anti-oxidant substances and/or increased consumption of oxidative substances may increase the prevalence and severity of asthma. Several studies showed that there is zinc deficiency in serum, hair and sputum of asthmatic patients (9-11). Zinc deficiency cause a decrease in level and activity of TH1 and an increase in level and activity of TH2 (12). Growth retardation, delayed wound healing, increased infections; chronic diarrhea and pubertal delay are complications of zinc deficiency (13). Many studies showed a positive relation between zinc consumption in mother and produce of asthma in children (grade C studies). But there was no relation between serum zinc levels in mother and cord blood with prevalence of asthma in children (10). However, despite several investigations of zinc trace elements in children with asthma, the relationship is not well understood.

The main purpose of this review study is to evaluate the zinc levels in serum and hair in children with asthma (primary outcome) with meta-analysis and our second goal was zinc supplement in children with asthma (secondary outcome) without meta-analysis. 

## Methods

To gather the articles, we searched in several databases include Google scholar, PubMed, Elsevier, Scopus and up-to-date. Key words were zinc, children, pediatric, asthma, deficiency and treatment. All studies which evaluated the zinc level and treatment in children with asthma until July 2019 were included. Because serum zinc levels are variable in patients and non-patients, we included articles that had a control group along with asthma patients. Animal studies excluded. Only English language articles were included. We did not have limitation time for the onset of research. Inclusion criteria include serum zinc level, hair zinc level, children with asthma, children with recurrent wheezing, zinc supplement therapy and compared with a control group. Exclusion criteria include adult age and did not have a control group. 

All studies were searched by at least two researchers. All abstracts were screened by an allergist and clinical immunologist and full papers were screened by an allergist and epidemiologist. Quality assessment was according to the following criteria; is a qualitative approach appropriate? Is the study clear in what it seeks to do? How defensible/rigorous is the research design/methodology? How well was the data collection carried out? Is the context clearly described? Were the methods reliable? Are the data’ rich’? Is the analysis reliable? Are the findings convincing? Are the conclusions adequate? Was the study approved by an ethics committee? Is the role of the researcher clearly described?

The data were analyzed using STATA Version 11. The mean difference with 95% confidence interval (95% CI) was used as an effect size and a random effect was used for pooled estimation. Heterogeneity was assessed by I2 values.

## Results

We did not find many articles. Out of 40 articles, 19 articles were excluded because they were written in languages other than English, either no access to full article or had no control group. Finally, 21 studies were included in the analysis. 15 articles evaluated serum zinc level in children with asthma in the world (table 1). 4 articles on hair zinc level and one article on nail zinc level in children with asthma (table 2). Also, one article evaluated zinc level in erythrocyte cells in children with asthma. Only 3 articles evaluated zinc supplement treatment in children with asthma (table 3). All of the studies used atomic absorption for the detection of zinc levels. As we found so few articles about the evaluation of zinc level in nail of children with asthma and zinc supplement therapy in children with asthma, we could not perform a meta-analysis. We did meta-analysis of serum and hair zinc levels in children with asthma.

**Table 1:** Characteristics of serum zinc levels in children asthma and control groups

**Author **	**Asthma(N), zinc level**	**Healthy (N), zinc level**	**Pv**	**Ages**	**Blood sample**
Abdulwahab14.	(40), 12.69±1.80 µmol/L	(40), 13.0±1.52 µmol/L	0.388	7-14 years old	Non fasting
Kocyigit15	(42),765.72±167.16 µg/L	(30), 775.74±106.38 µg/L	0.812	2-14 years old	Fasting
el-Kholy16	(40), 70.3±13.2 µg/dl	(20), 88.4 ±11.0 µg/dl	0.001	2-12 years old	-
Bilan17	(50), 20.12±10.14 µg/dl	(50),25.20±8.95 µg/dl	0.009	2-18 years old	Fasting
Ermis18	(41), 70.6±8.3 µg/dl	(30), 78.3±9.2 µg/dl	0.01	Mean age 7.6±1.8 years old	-
Khanbabaee 19	(150), 70.5±22.6µg/dl	(100), 80.9±16.9 µg/dl	0.001	3-149 months	-
Di Toro20	(22), 16.2±0.5 µmol/L	(19), 16.2±0.8 µmol/L	>0.05	20-168 months	Fasting
Ghaffari21	(175), serum 83.08±44.96 µg/dl	(165), 85.06±20.35 µg/dl	0.752	3-19 years old	Fasting
Kakarash22	(50), 70.02µg/dl	(50), 84.04µg/dl	<0.01	1-12 yreas old	-
Wagdy23	(38), 249.63±93.97 µg/dl	(17), 148.47±60.924 µg/dl	0.00	24-101 months	-
Soliman24	(50), 49.1±8.5 µg/dl	(30), 95±10.8 µg/dl	0.001	3-12 years old	-
Ariaee4	(49), 831±120 µg/L	(24), 910±116 µg/L	0.001	10-50 years old	-
Behmanesh25	(80), 97.18±23.59µg/dl	(80), 93.3±25.58 µg/dl	0.406	Mean 6.07±2.67 years old	-
Andino26	(24), 759µg/L	(2), 910 µg/L	0.011	8-18 years old	-
Uysalol27	(46), 0.70±0.13	(43), 0.73±0.15	0.186	3-24 months	fasting≥4h

**Table 2:** Characteristics of nail and hair zinc levels in children asthma and control groups

**Author **	**Asthma(N), zinc level**	**Healthy (N), zinc level**	**Pv**	**Ages**	**Blood sample**
Carneiro28	(40),nail zinc over 143.71 µg/gr in 82%	(125),nail zinc over 143.71 µg/gr in 95.2 %	0.01	12-18 years old	-
el-Kholy16	(40),167.5 ±23.0 µg/gm in hair	(20),194.5 ± 18.6 µg/gm in hair	0.001	2-12 years old	-
Di Toro20	(22), hair 100±8 µg/gr	(19), hair 147±9 µg/gr	<0.05	20-168 months	Fasting
Razi29	(65), hair 162.43 ±91.52 µg/gr	(65), 236.38±126.44 µg/gr	0.001	35.46±16.33 months	-
Tahan30	(34), hair 4-85 µg/gr	(14), hair 111-175 µg/gr	0.001	1-3 years old	-

**Table 3:** Characteristics of zinc supplement treatment in children with asthma

**Author**	**Intervention**	**Control**	**Pv**	**Ages**	**Blood sample**	**Detection method**
Ghaffari9	(155), 50 mg daily for 8 weeks Serum zinc <70 µg/dl	(145), only asthma treatment Serum zinc <70 µg/dl	0.00	5-15 years old	Fasting	Atomic
Rerksuppaphol31 , acute asthma	(21), 30 mg/daily zinc treatment Serum zinc 63.8±17.4	(21), placebo	0.693	2-16 years old	Fasting, nonfasting	Atomic
Biltagi32	(76), zinc treatment 15mg/day	Asthma control group ,	0.001	7-10 years old	-	Improved in PFT, C-ACT.

**Figure 1:**
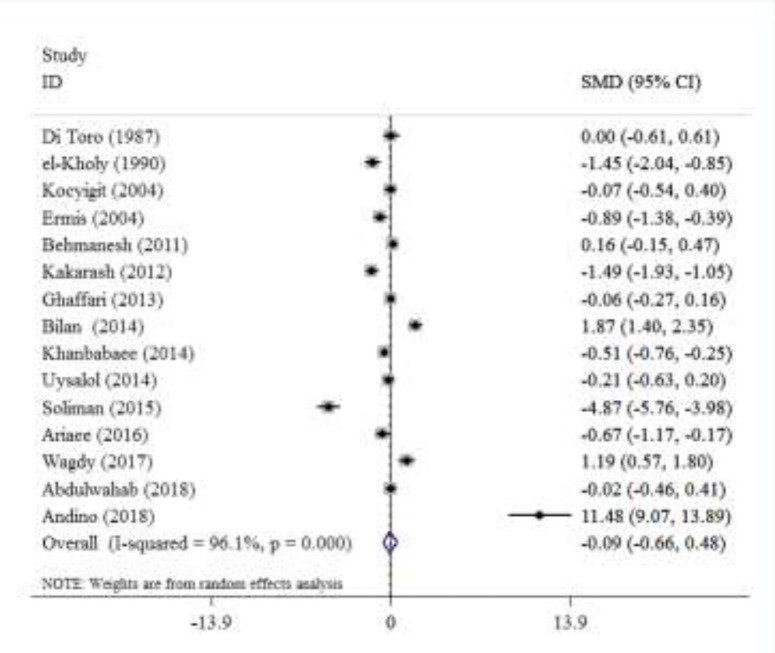
Frost plot of standard mean differences for serum Zinc level in asthmatic patients compared to the control group

**Figure 2:**
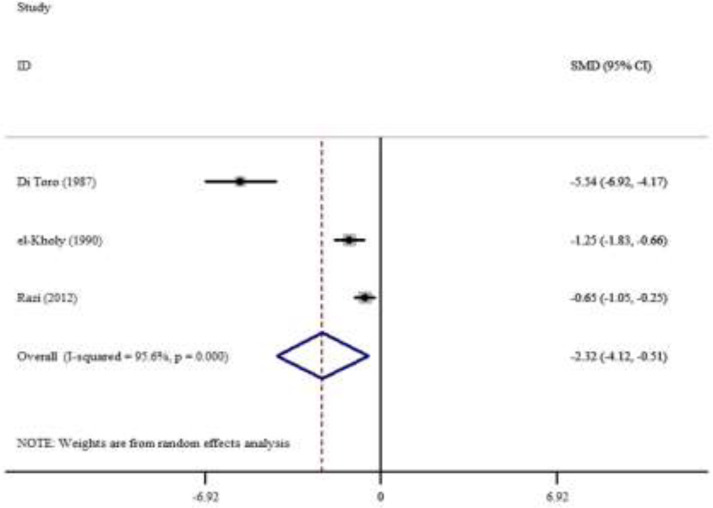
Frost plot of standard mean differences for hair Zinc level in asthmatic patients compared to the control group

We found only one study of zinc level in erythrocyte of children with asthma. Yilmaz et al. studied erythrocyte zinc level in children with asthma. The age range was between 8-164 months. 67 cases of asthma and 45 healthy children were evaluated. There was no significant difference in erythrocyte zinc levels between children with asthma (1215.8±145.1µg/dl) and healthy group (1206.9±119.5µ/dl) (P=0.472) (8). The meta-analysis of studies showed that there was not a significant difference between standard mean differences of serum Zinc level in asthmatic patients compared to the control group. There was heterogeneity (I2=96.1%, P=0.000) between these studies that were included in this meta-analysis (fig 1). Figure 2 shows that there was a significant difference between standard mean differences of hair zinc level in asthmatic patients compared to the control group. There was heterogeneity (I2=95.6%, P=0.000) between these studies that were included in this meta-analysis. 

## Discussion

Oxidative factors have variable effects on asthma and anti-oxidative has different effect on asthma. Enzymatic anti-oxidants require zinc, copper, selenium and others for doing (33, 34). Nutrition is one of the environmental factors that may influence children with asthma. Mineral nutrients such as zinc could be important in asthmatic patients (8, 26). The reasons for the lower serum zinc in asthmatic patients are zinc redistribution to hepatocytes, increased demand of zinc, decreased zinc reserves in respiratory cell due to increased oxidative stress (26). Although more studies evaluated serum zinc level but some studies evaluated hair or nail zinc level because they believed that hair or nail zinc level could reflect body zinc levels (30), but some studies do not confirm that (17, 20, 35). The cutoff for zinc deficiency is not the same in these studies, therefore, the prevalence of zinc deficiency is varied (21, 19, 20, 31). 

Although most studies (9 out of 15) show that there is a difference in mean serum zinc levels between asthmatic children and healthy control group (4, 16-19, 22-23, 26). Only in one study, the mean serum zinc level was higher in children with asthma than the healthy group (23), while in other studies, the mean serum zinc levels were lower in children with asthma than control group patients (4,16-19, 22, 26). Our meta-analysis revealed that there is not a significant difference in the mean serum zinc levels between asthmatic children and healthy group, probably due to the heterogeneity of these studies. It seems that the sample size was low in many studies; this perhaps has affected the results. 

In 6 studies, there is no significant difference in the mean serum zinc level between asthmatic children and healthy control group (1, 15, 20-21, 25, 27). Except in one study, the mean serum zinc level was higher in asthmatic children than control group (25), other studies showed lower mean serum zinc in asthmatic children than control group subjects (1, 15, 20, 21, 27). In Goldey’s study, there was no significant difference of serum and hair zinc level between children with asthma (n=29) and healthy children (n=21) ages between 6 to 20 years old (36). 

Is there a relationship between mean zinc serum levels with the severity of children`s asthma? In response to this question, Abdulwahab et al. found that there was not a significant relationship between serum zinc level and the severity of children asthma (14). the same result was seen in Bilan`s and Wagdy et al.`s studies (17, 23). While Khanbabaee et al. showed there was a significant difference between serum zinc deficiency and severity of asthma in children. They stated that serum zinc deficiency was more common in patients with severe persistent asthma than mild or moderate asthma (P=0.001) (19). Also in Kakarash`s study, serum zinc level was lower in children with severe asthma (P=0.01) but there was no significant difference with duration of asthma (P=0.69). Serum zinc level was lower in subjects who used corticosteroids than those who did not (P=0.02) (22). Khanbabaee and Kakarash stated that the mean serum zinc levels are significantly lower in children with severe asthma (19, 22), Soliman`s study revealed that mean serum zinc levels was lower in moderate asthma than mild asthmatic patients (P=0.00) (24). 

Ariaee and Behmanesh’ studies showed that serum zinc level was not related with the severity of asthma (4, 25). Whereas Uysalol reported that serum zinc levels were significantly lower in children with recurrent wheezing than other patients (0.63±0.1) vs 0.72±0.12 respectively, P=0.07) (27). Overall, the association between mean serum zinc levels and the severity of asthma in children is not clear. We did not do the meta-analysis of serum zinc level with severity of asthma, because there were not enough articles and more heterogeneity of them. 

In our previous study, we showed that there was no significant difference of serum zinc levels between male and female asthmatic patients and healthy control group (21). Out of all the studies that evaluated serum zinc levels in childhood asthma, one study evaluated serum zinc levels in children and adults (10-50 years old), it is not statically significant in altering our results(4). We found one study investigating the relationship between nail zinc concentration and childhood asthma (28), the study reported that the prevalence of asthma was lower in children with higher nail zinc concentration (28). Tahan set 100 µg/g as his cutoff point and values lower than this were considered to be deficient. In this study, hair zinc levels were significantly lower in children with recurrent wheezing than healthy cases (30). Di Toro et al. revealed that the prevalence of hair zinc deficiency was 35% in children with allergy. 

We found a few studies on zinc levels in hair and nail in children with asthma, a meta-analysis performed on the zinc level in hair and children with asthma (figure 2). Our analysis showed that the hair zinc level was significantly lower in children with asthma than healthy group. But more studies with bigger sample size need to confirm it. One study evaluated erythrocyte zinc levels and stated that there is no significant relationship between the erythrocyte zinc levels in asthmatic subjects and asthma duration, severity, and asthma control level (8). 

**Zinc supplement therapy: **Zinc supplementation has varied effects on children with asthma. Biltagi et al. showed that zinc improved pulmonary function tests, childhood asthma control test (c-ACT) and pulmonary inflammatory markers in children with moderate persistent asthma (P=0.001). Sputum ECP, WBC count and eosinophil ratio significantly decreased in children with persistent moderate asthma (P=0.001). Administering omega-3 fatty acids (1000 mg/day), zinc (15mg/day) and vitamin C (200mg/day) together proved more efficient due to higher antioxidant effects. They did not mention if their subjects were zinc-deficient at baseline or not (32). In a clinical trial study by Ghaffari et al. zinc supplementation (50 mg daily for 8 weeks) in children with asthma, improved both clinical manifestations and pulmonary function test compared to control group subjects. The difference between this study and Biltagi’s study is that we used double dose of zinc and our patients were zinc-deficient at baseline (9). 

In another clinical trial study, 57 percent (24 out of 42) of children with asthma attack had serum zinc deficiency. Both groups were treated with classic asthma attack drugs, one group was given zinc supplement (30 mg/day elemental zinc for 4 days) and another group was given placebo. After treatment, serum zinc levels increased significantly in both groups but there was no difference between the two groups (P=0.238). The average number of admission days was not significantly different between the two groups (P=0.504). Pediatric respiratory assessment measure (PRAM) significantly improved in case group patients than the control in the first 48 hours, but there was no difference at the end of the study between two groups (96 hours) (31). All the clinical trials that were mentioned found that zinc supplementation (regardless of dosage) improved the clinical manifestations of children with asthma. Because there are few studies investigating this matter, more studies need to be performed to confirm the positive effects of zinc in children with asthma.

In conclusion we revealed that the mean serum zinc level is not significantly different in children with asthma and healthy control group. It seems that there is no relationship between mean serum zinc levels and severity of asthma in children; although the studies are heterogeneous due to difference in sample size, normal cutoff of zinc level and age range. We need more studies with bigger sample sizes and powerful methodology to confirm the relationship between the mean serum zinc levels in children with asthma. It seems there is more association between hair zinc concentrations with children asthma. Even though these studies do not have powerful methodologies; zinc supplementation could be effective in the prevention and treatment of asthma in children as an additive.
